# Relationship between Markers of Gut Barrier Function and Erythrocyte Membrane PUFAs in Diarrhea-Predominant IBS Patients Undergoing a Low-FODMAP Diet

**DOI:** 10.3390/nu16162706

**Published:** 2024-08-14

**Authors:** Michele Linsalata, Antonia Ignazzi, Benedetta D’Attoma, Giuseppe Riezzo, Domenica Mallardi, Antonella Orlando, Laura Prospero, Maria Notarnicola, Valentina De Nunzio, Giuliano Pinto, Francesco Russo

**Affiliations:** 1 Functional Gastrointestinal Disorders Research Group, National Institute of Gastroenterology IRCCS “S. de Bellis”, 70013 Castellana Grotte, Italy; michele.linsalata@irccsdebellis.it (M.L.); antonia.ignazzi@irccsdebellis.it (A.I.); benedetta.dattoma@irccsdebellis.it (B.D.); giuseppe.riezzo@irccsdebellis.it (G.R.); domenica.mallardi@irccsdebellis.it (D.M.); antonella.orlando@irccsdebellis.it (A.O.); laura.prospero@irccsdebellis.it (L.P.); 2 Laboratory of Nutritional Biochemistry, National Institute of Gastroenterology IRCCS “S. de Bellis”, 70013 Castellana Grotte, Italy; maria.notarnicola@irccsdebellis.it (M.N.); valentina.denunzio@irccsdebellis.it (V.D.N.); giuliano.pinto@irccsdebellis.it (G.P.)

**Keywords:** dietary carbohydrates, fatty acids, omega-3, fatty acids unsaturated, fermentation FODMAPs, intestinal permeability, irritable bowel syndrome, zonulin

## Abstract

Many patients with irritable bowel syndrome (IBS) have a compromised intestinal barrier associated with low-grade inflammation. Polyunsaturated fatty acids (PUFAs) are potential mediators of inflammation: omega-6 PUFAs are pro-inflammatory, while omega-3 PUFAs are antioxidant and anti-inflammatory. Zonulin is a potential biomarker for small intestinal permeability (s-IP). This study investigated the relationship between PUFAs and gastrointestinal (GI) barrier integrity in IBS patients with predominant diarrhea (IBS-D). We evaluated GI barrier function indicators in the urine and bloodstream and erythrocyte membrane PUFA composition in 38 IBS-D patients (5 men, 33 women, 44.11 ± 1.64 years), categorized at baseline by fecal zonulin levels into high (≥107 ng/mL, H-FZ) and normal (<107 ng/mL N-FZ) groups. Evaluations were conducted prior to and following a 12-week diet low in FODMAPs (LFD). At baseline, H-FZ patients had s-IP significantly higher than the reference value, lower *n*-3 PUFAs levels, and higher *n*-6/*n*-3 PUFAs and arachidonic acid (AA) to eicosapentaenoic acid (EPA) ratios than N-FZ. After LFD, H-FZ patients showed significant increases in *n*-3 PUFAs levels; decreases in *n*-6 PUFAs, *n*-6/*n*-3 PUFAs and AA/EPA ratios; and improved s-IP. The *n*-6/*n*-3 PUFAs ratio positively correlated with fecal zonulin levels in all subjects. These findings highlight the relationship between PUFAs and the intestinal barrier, suggesting their role in IBS-D pathophysiology and confirming the positive effects of LFD in managing IBS-D.

## 1. Introduction

Irritable bowel syndrome (IBS) is a prevalent functional gastrointestinal (GI) disorder marked by chronic abdominal pain or discomfort and altered bowel habits over an extended period [1]. According to the Rome IV diagnostic criteria, IBS can be categorized into four types: IBS with constipation (IBS-C), IBS with diarrhea (IBS-D), IBS with mixed bowel habits (IBS-M), and unclassified IBS (IBS-U) [2].

IBS is more frequently diagnosed in women, who are 1.5 to 3 times more likely to develop the condition compared to men [3]. This gender disparity is thought to stem from factors such as hormonal fluctuations, heightened pain sensitivity, psychological stress, and anxiety. Additionally, emerging research points to differences in gut microbiota and genetic predispositions as contributing factors. Although the underlying mechanisms remain complex, the evidence consistently indicates a higher prevalence of IBS in women.

IBS etiology is multi-factorial, involving neurological and immune system disturbances and changes in the intestinal microbiota and barrier function [3]. The intestinal barrier is a defense mechanism, shielding the host from pathogens and toxins while facilitating nutrient absorption [4]. When this barrier is compromised, increased small intestinal permeability (s-IP) can occur, particularly in IBS-D patients and may lead to low-grade immune activation [5]. This increased permeability allows toxins and bacteria to enter the bloodstream, triggering immune responses and sustaining inflammation [6]. This disrupts gut sensory and motor functions, resulting in common IBS symptoms such as abdominal pain, bloating, and diarrhea [1].

Recent research has identified polyunsaturated fatty acids (PUFAs) as potential mediators of intestinal inflammation. Omega-6 PUFAs are associated with pro-inflammatory effects, while omega-3 PUFAs are recognized for their anti-inflammatory and antioxidant properties. Active metabolites of these PUFAs, such as arachidonic acid (AA) and eicosapentaenoic acid (EPA), serve as biomarkers of inflammation in metabolic disorders [7]. EPA, in particular, has demonstrated anti-inflammatory effects by partially inhibiting the nuclear factor-kB pathway in adipocytes [8]. While human studies on the relationship between PUFAs and the intestinal barrier are limited, preclinical studies suggest that PUFAs may influence intestinal permeability by modulating tight junctions (TJ) [9,10]. Notably, an increase in *n*-3 PUFAs through a Mediterranean diet has been shown to improve intestinal barrier dysfunction. A helpful approach to exploring the connection between PUFAs and inflammation involves analyzing blood biomarkers of fatty acid (FA) intake, such as the FA composition of red blood cell membranes. The lipid profile of erythrocyte membranes can provide a systemic nutritional “snapshot”, reflecting interactions among genetic, metabolic, and dietary factors [11].

Non-invasive assessments of intestinal barrier function can be conducted using urinary assays of non-absorbable sugars like lactulose (Lac), mannitol (Man), and sucrose (Suc). An elevated Lac/Man ratio suggests s-IP dysfunction, while Suc levels indicate gastroduodenal permeability [12]. Although effective, this multi-sugar test requires overnight fasting and urine collection, which can be cumbersome. Consequently, recent research has focused on alternative biomarkers, such as zonulin—a protein that regulates TJ permeability. Serum and fecal zonulin levels are emerging as promising non-invasive markers of intestinal permeability, with fecal zonulin particularly indicative of barrier leakage [13,14]. Furthermore, intestinal fatty acid-binding protein (I-FABP) and diamine oxidase (DAO), which enter the bloodstream when the intestinal barrier’s integrity is compromised, are being studied for their potential as indicators of barrier integrity [15].

Dietary interventions, particularly those that restrict fermentable oligosaccharides, disaccharides, monosaccharides, and polyols (FODMAPs), have shown promise in alleviating IBS symptoms and enhancing intestinal barrier function [16,17]. A recent study observed symptom improvement and decreased inflammation markers in IBS-D patients following a low-FODMAP diet (LFD) [18]. Another study [19] reported minimal inflammation in IBS-D, with significant reductions in prostaglandin E2 levels linked to PUFAs like AA after an LFD.

It is hypothesized that IBS-D patients may exhibit alterations in the PUFA composition of erythrocyte membranes due to impaired intestinal permeability. Moreover, a 12-week LFD might modify the PUFA profile of erythrocyte membranes and improve GI barrier integrity.

Based on this hypothesis, our research aimed to explore the relationship between PUFAs and GI barrier integrity in individuals with IBS-D following a 12-week LFD. We assessed intestinal barrier function and integrity by measuring the urinary excretion of Lac, Man, and Suc as markers of GI permeability, as well as the circulating concentrations of I-FABP and DAO. Additionally, we evaluated the extent of inflammation in IBS-D by measuring interleukins 6, 8, 10 (IL-6, IL-8, IL-10) and Tumor Necrosis Factor-alpha (TNF-α). Finally, this study sought to determine whether a 12-week LFD led to changes in these parameters.

## 2. Materials and Methods

### 2.1. Patient Recruitment

In this prospective study, conducted from January 2021 to February 2022, patients were recruited from the outpatient clinic of the Functional Gastrointestinal Disorder Unit at the National Institute of Gastroenterology “S. de Bellis” in Puglia, Italy, based on the Rome IV diagnostic criteria. Each patient underwent a physical examination, and baseline urine and serum samples were collected.

Inclusion criteria were as follows: patients aged 18–65 who had experienced IBS-D-like symptoms for a minimum of 12 weeks, with a mean score of >75 on the IBS-Severity Scoring System (IBS-SSS), and demonstrated a specific stool pattern as defined by Schmulsson et al. [20]. Recent diagnostic tests included blood tests (liver function, thyroid function, reactive protein C) within the previous three months, stool occult blood tests (three determinations), stool culture, and tests for parasites. Gastroscopy and colonoscopy were also required, and celiac disease was excluded through testing for tissue transglutaminase and anti-endomysium antibodies. Only patients who tested negative for HLA-DQ2/HLA-DQ8 were included to rule out gluten-sensitive diarrhea unrelated to celiac disease [21]. Participants were also required not to have followed restrictive diets (e.g., low FODMAP, gluten-free, or vegan diets) before joining this study.

Exclusion criteria included constipation, post-infectious IBS, giardiasis, pregnancy, previous abdominal surgery, metabolic and endocrine disorders, hepatic, renal, or cardiovascular diseases, fever, intense physical activity, secondary causes of intestinal atrophy, history of malignancy, recent use of antibiotics, probiotics, SSRIs, other antidepressants, and medications known to cause abdominal pain, as well as the use of IBS-specific drugs within the two weeks prior to evaluation.

Reasons for study discontinuation were documented in the case report form and could include adverse events, ineligibility to continue, withdrawal of consent, loss to follow-up, or other reasons.

All participants provided written informed consent for data collection. This study was registered at ClinicalTrials.gov (NCT03423069), with the last data access on 22 March 2022. The research was approved by the local Scientific Committee and the Institutional Ethics Committee of the IRCCS Oncological Hospital—John Paul II Cancer Institute, Bari, Italy (N. 274/E.V.).

### 2.2. Study Design

The study design consisted of three visits. During the first visit (Baseline), participants provided informed consent after being informed about this study’s objectives and underwent a gastroenterological examination. Qualified nutritionists evaluated the participants’ lifestyle, dietary habits, and any health issues. Participants were instructed to maintain their regular diet and keep a daily food diary for seven days, recording stool characteristics, medication use, physical activity, and food intake to estimate energy expenditure.

At the second visit (Attribution to diet), anthropometric measurements were taken. Participants completed the IBS-SSS questionnaire [22], and patients with a whole score above 75 were enrolled in this study. The food diaries from the previous week were reviewed to reassess the inclusion and exclusion criteria. Each participant was provided with an individualized diet and asked to continue keeping a daily diary tracking food, sexual activity, exercise, drugs, and stool forms. Stool, urine, and serum samples were also collected for analytical measurements and sugar absorption testing (SAT).

During the third visit (Final visit), the symptoms and dietary questionnaires completed over the 12-week diet period were collected. Participants completed the IBS-SSS scale and the IBS Diet Adherence Report Scale—Food Diary (IDARS) to evaluate dietary adherence. Finally, anthropometric and biochemical measurements were repeated following the same procedures as during the second visit.

### 2.3. Intervention Diet

During visit 2, an individualized LFD was prescribed, based on a review of the patients’ food diaries and consultations with nutritionists. The LFD is designed to restrict the intake of fermentable oligosaccharides, disaccharides, monosaccharides, and polyols [23]. Dedicated software (Nutrigeo 8.6.0.0, Progeo Medical, Centobuchi di Monteprandone, AP, Italy) was employed to evaluate daily macronutrient intake, ensuring a balanced distribution of 20% protein, 30% fat, and 50% carbohydrates. The diet formulation followed the methods outlined in previous research [17]. Each patient was provided with a detailed weekly menu, including breakfast, lunch, dinner, and two light snacks (one in the afternoon and one in the mid-morning). They also received a brochure that outlined permissible foods, prohibited items, and recommendations for reducing certain foods according to Monash University guidelines [24]. Fiber intake was maintained at adequate levels, and alcohol consumption was advised against.

### 2.4. Symptom Profile

The symptom profile was evaluated using the validated GI Symptom Questionnaire, specifically the IBS-SSS [22]. This comprehensive tool measures the score of IBS symptoms by assessing five aspects on a visual analog scale. These aspects include “Severity of abdominal pain”, “Frequency of abdominal pain”, “Severity of abdominal distension”, “Dissatisfaction with bowel habits” and “Impact of symptoms on quality of life”. Each aspect is rated on a scale from 0 to 100. Patients indicate their feelings by selecting a point on the line for items 1 through 4, with the distance from zero determining the score.

For the fifth item, patients report the percentage of days in which they experience abdominal pain; this response is multiplied by ten to convert it to a 0–100 scale. The total score, ranging from 0 to 500, is obtained by summing the scores of the five items. Scores classify cases as “mild” (75 to 175), “moderate” (>175 to 300), or “severe” (over 300). Typically, healthy individuals score below 75, and patients are considered in remission with scores under 75. Bowel habits were documented calculating the Bristol stool form scale [25].

### 2.5. Assessment of Nutrient Intake

Patients were instructed to maintain a food diary before this study and during the dietary intervention to evaluate calorie intake and expenditure. Nutritionists reviewed these diaries, documenting the types and quantities of food consumed (in grams) for breakfast, lunch, dinner, and snacks, along with the nature and duration of physical activity [17]. The information was analyzed using specific software (Progetto Dieta v. 2.0, accessed at http://www.progettodieta.com; accessed on 22 April 2023). This software calculated the daily intake of carbohydrates, proteins, lipids, dietary fiber, and the percentage of alcohol consumption. Additionally, it provided the daily energy intake and expenditure, expressed in kilocalories.

### 2.6. Anthropometric Profile Assessment

The anthropometric measures listed below were assessed: weight, height, and waist circumference. Weight and height were measured using a SECA mod. 700 mechanical column scale and a SECA mod. 220 stadiometer (INTERMED S.r.l., Milan, Italy), respectively, to calculate BMI (kg/m²). A SECA mod. 201 tape measure was used to determine waist circumference.

### 2.7. Intestinal Barrier Function Biomarkers and Integrity and the Indices of Inflammation

Biomarker assessments were carried out before and after the dietary intervention. Stool samples from participants were stored at −80 °C within 12 h of collection. Serum and stool zonulin levels were measured with Immunodiagnostik AG’s (Bensheim, Germany) ELISA kits. Fecal zonulin levels below 107 ng/mL were deemed normal according to the manufacturer’s guidelines. Based on fecal zonulin concentrations, patients were categorized into two groups: those with high fecal zonulin levels (H-FZ) and those with normal levels (N-FZ).

Serum levels of DAO and I-FABP were measured employing ELISA kits from Thermo Fisher Scientific (Waltham, MA, USA) and Cloud-Clone Corp (Houston, TX, USA), respectively. Additionally, serum levels of IL-6, IL-8, IL-10, and TNF-α evaluated with ELISA kits provided from Elab Science Biotechnology Inc. (Houston, TX, USA).

### 2.8. Sugar Absorption Test (SAT)

All patients underwent an s-IP assessment using a sugar absorption test (SAT) following an overnight fast. Initially, urine samples were collected to check for the presence of natural sugars. Participants then took a solution with 10 g of lactose (Lac), 5 g of mannitol (Man), and 40 g of sucrose (Suc) dissolved in 100 mL of liquid. After consuming the solution, urine samples were collected over a 5-h period, and the total volumes were measured and recorded. An aliquot of 2 mL from each sample was stored at −80 °C until analysis.

Chromatographic analysis was performed according to previously described methods [12] to determine urinary levels of Lac, Man, and Suc. The percentages of Lac (%Lac), Man (%Man), and Suc (%Suc) expelled relative to the amount ingested were calculated, and the Lac/Man ratio was computed for each sample. Ratios exceeding 0.03 were considered indicative of impaired absorption [26].

### 2.9. Fatty Acids Analysis

FA extraction and the transesterification of lipids into fatty acid methyl esters (FAMEs) were performed using an automated protocol (Robot LNG-R1, Lipinutragen-Tecan, Bologna, Italy). Briefly, whole blood collected in EDTA tubes was centrifuged at 4000× *g* for 5 min at 6 °C. Mature erythrocytes, aged at least three months, were isolated based on density. After removing the plasma, the cells were lysed osmotically, and the resulting membrane pellets were used for phospholipid extraction via the Bligh and Dyer method [27]. The organic layer containing chloroform was separated and evaporated using a centrifugal evaporator (Waltham, MA, USA’s Thermo Fisher Scientific). The FAMEs were then transesterified with potassium hydroxide. Following extraction, the esterified fatty acids were re-suspended in *n*-hexane and analyzed using gas chromatography with an autosampler, a split/splitless injector, a flame ionization detector (FID), and a hydrogen gas generator (Thermo Fisher Scientific, Milan, Italy) as described previously [28]. FAMEs quantification was performed with a standard mixture (Supelco 37-Component FAMEs Mix, Sigma-Aldrich, Milan, Italy). Reference interval values for PUFA composition in erythrocyte membranes from a healthy population are provided in the results section [29].

### 2.10. Statistical Analysis

All results are presented as the means ± SEM. To avoid assumptions of normal distribution, non-parametric tests were used. The Wilcoxon rank-sum test was applied to detect differences in biochemical parameters and IBS-SSS questionnaire scores before and after LFD. The Mann–Whitney test was employed for comparison of the two subgroups at both the start and end of the diet. Spearman rank correlation was used to assess the relationship between fecal zonulin levels and other variables. A significance level of 5% was applied. Statistical analyses were performed using Stata Statistical Software, release 9 (2005; College Station, TX, USA: Stata Corp.).

## 3. Results

### 3.1. The Number of Patients, Their Anthropometric Traits, and the Intervention Diet

**Figure 1:**
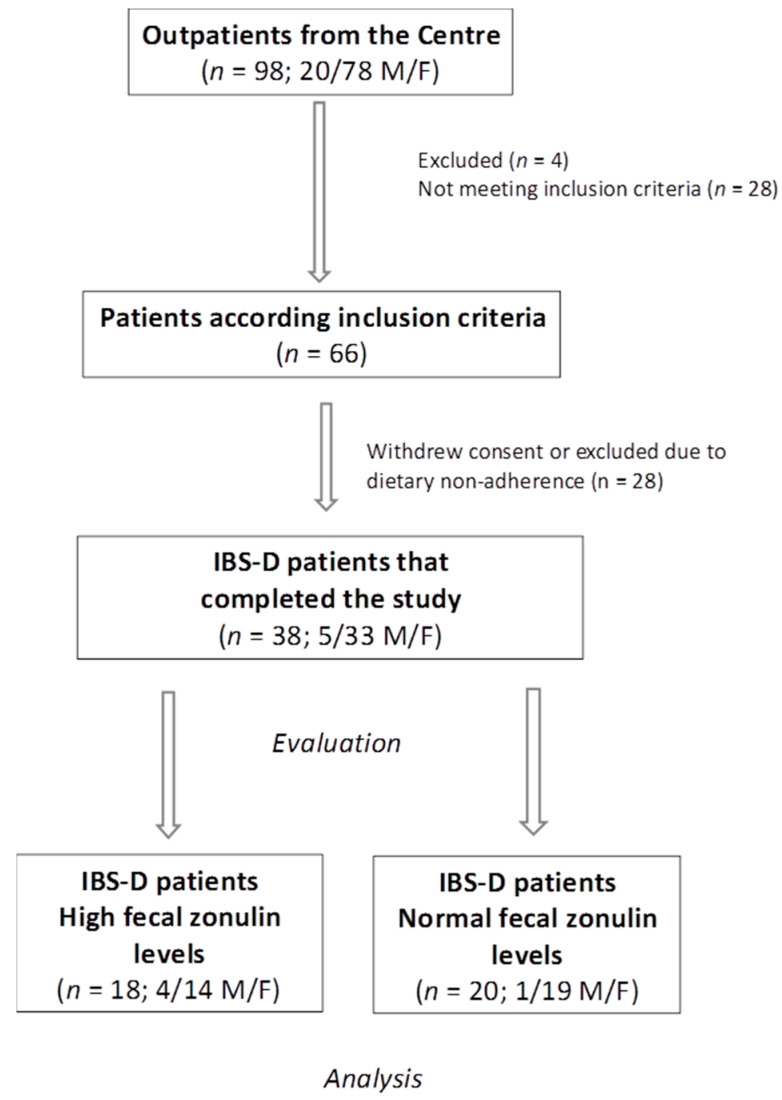
The flow of participants through this study. IBS-D = irritable bowel syndrome with prevalent diarrhea. F = females. M = males.

The patient flow throughout this study is shown in Figure 1. A total of 98 patients with IBS-D were initially recruited, consisting of 20 males (M) and 78 females (F). The reasons for exclusion were as follows: 4 patients were removed for various reasons, 28 did not fit the requirements for inclusion, and another 28 either refused to participate or were excluded due to dietary non-compliance. Ultimately, 38 patients (5 males and 33 females; mean age = 44.11 ± 1.64 years) completed the 12-week low-FODMAP diet (LFD) study. Among these 38 patients: 20 patients (52.6%, including 19 females and 1 male; mean age = 45.65 ± 2.25 years) had normal fecal zonulin levels (<107 ng/mL, N-FZ). Eighteen patients (47.4%, including 14 females and 4 males; mean age = 42.39 ± 2.47 years) had elevated fecal zonulin levels (≥107 ng/mL, H-FZ).

Table 1 presents the patients’ anthropometric characteristics at the start and end of the diet. Significant decreases (*p* < 0.05) at the end of testing were observed for weight, BMI, and waist circumference compared to the start of this study, both in the total population and the two subgroups considered. Patients’ daily nutritional information before and after LFD has already been described elsewhere [18].

### 3.2. The Symptom Profile

In the IBS-D patient group as a whole, the total IBS-SSS score showed a significant decrease of 49% after 12 weeks on the low-FODMAP diet (269.0 ± 12.0 vs. 137.4 ± 14.6; pre vs. post diet; *p* < 0.0001). Initially, the H-FZ and N-FZ subgroups’ symptom profiles did not differ significantly from each other. However, both subgroups experienced substantial improvements in their IBS-SSS scores following the diet. Specifically, in the H-FZ subgroup, the total IBS-SSS score decreased by 53.6% (123.2 ± 15.4 vs. 265.9 ± 15.5; *p* = 0.0001). In the N-FZ subgroup, the score was reduced by 44.7% (150.3 ± 24.1 vs. 271.8 ± 18.4; *p* < 0.0001). Between the H-FZ and N-FZ subgroups, there were no significant differences in the IBS-SSS total scores at the conclusion of this study, indicating that the dietary intervention led to similar improvements in IBS symptoms regardless of initial fecal zonulin levels.

### 3.3. Fecal and Serum Zonulin Levels

**Figure 2:**
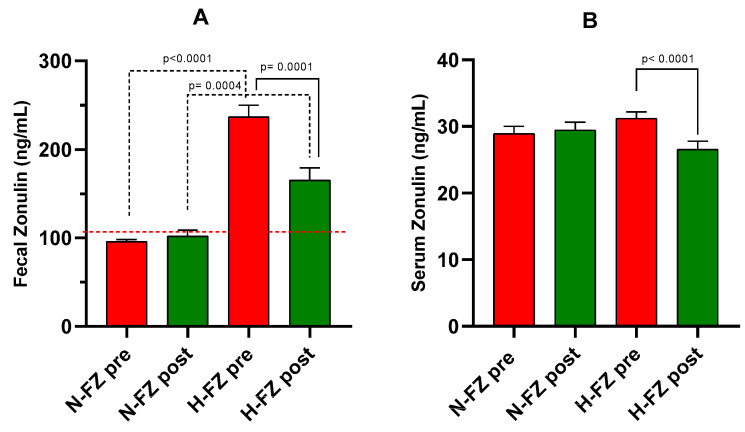
Zonulin levels in IBS-D patients. (**A**): Fecal zonulin levels. (**B**): Serum zonulin levels. Data are presented for measurements taken before (pre) and after (post) 12 weeks of a diet low in FODMAPs (LFD), categorized by baseline fecal zonulin levels as normal (N-FZ) or high (H-FZ). Data are indicated as the means ± SEM. The Wilcoxon rank-sum test (solid line) was performed to compare data pre- and post the diet. The Mann–Whitney test (black dotted line) was utilized to compare the two subgroups before and after the diet. Differences are assumed significant at *p* < 0.05. The red dotted line represents the cutoff value.

In the overall group of IBS-D patients, fecal zonulin levels decreased significantly by approximately 16% from baseline (162.6 ± 13.1 ng/mL) to the end of LFD (132.0 ± 8.9 ng/mL, *p* = 0.007). Patients were divided based on their baseline fecal zonulin levels: 20 out of 38 patients (52.6%) had levels below 107 ng/mL (N-FZ), while 18 out of 38 patients (47.4%) had levels at or above 107 ng/mL (H-FZ). Figure 2A displays fecal zonulin levels for these two subgroups. In the N-FZ subgroup, fecal zonulin levels after the LFD were similar to baseline values (95.8 ± 2.5 ng/mL vs. 102.1 ± 6.7 ng/mL). In the H-FZ subgroup, there was a significant reduction of 30.2% in fecal zonulin levels after the diet (165.3 ± 13.8 ng/mL), compared to baseline levels (236.9 ± 12.9 ng/mL, *p* = 0.0001). Despite this decrease, fecal zonulin concentrations in the H-FZ subgroup remained significantly higher than those in the N-FZ subgroup (*p =* 0.0004).

Regarding serum zonulin, the overall group of IBS-D patients exhibited a significant decrease after the diet (28.5 ± 0.9 ng/mL vs. 29.9 ± 0.7 ng/mL, *p* = 0.044). Specifically, in the H-FZ subgroup, serum zonulin levels decreased significantly (26.5 ± 1.2 ng/mL vs. 31.2 ± 0.9 ng/mL, *p* < 0.0001). On the opposite, in the N-FZ subgroup, serum zonulin levels remained stable before and after the diet (29.4 ± 1.1 ng/mL vs. 28.9 ± 1.1 ng/mL). No significant differences in serum zonulin levels were observed between the N-FZ and H-FZ subgroups either before or after the LFD (Figure 2B).

### 3.4. Gastrointestinal Permeability

The s-IP of patients with IBS-D was assessed using SAT before and after the 12-week LFD. Following the diet, the percentages of lactulose (% Lac) and mannitol (% Man) were significantly reduced compared to pre-diet levels (0.26 ± 0.03 vs. 0.43 ± 0.07, *p* < 0.0001; and 12.48 ± 0.55 vs. 13.71 ± 0.52, *p* = 0.0048, respectively). Consequently, the Lac/Man ratio decreased by 31% (0.020 ± 0.002 vs. 0.030 ± 0.004, *p* < 0.0001). There was also a significant reduction in the percentage of urinary sucrose excreted after the diet (0.18 ± 0.04 vs. 0.25 ± 0.05, *p* = 0.012). Among all IBS-D patients, % Lac positively correlated with fecal zonulin levels (r = 0.42, *p* = 0.009).

**Figure 3:**
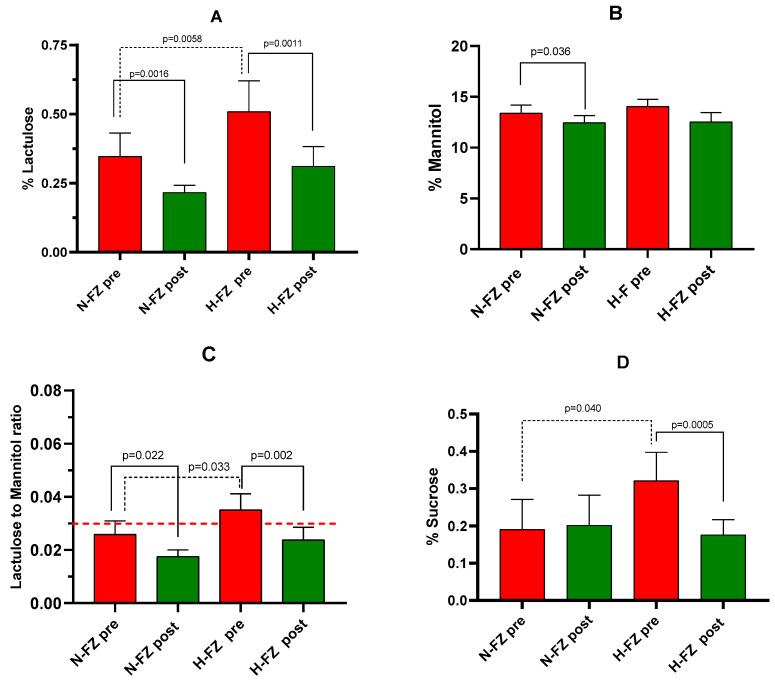
Gastrointestinal permeability assessed by the sugar absorption test. (**A**): % lactulose (Lac). (**B**) % mannitol (Man). (**C**): Lac/Man ratio. (**D**): % sucrose. Data are shown for measurements taken before (pre) and after (post) 12 weeks of a diet low in FODMAPs (LFD) in IBS-D patients, categorized by their baseline fecal zonulin levels as either normal (N-FZ) or high (H-FZ). Data are provided as the means ± SEM. The Wilcoxon rank-sum test (solid line) was used for comparison of data before and after diet. The Mann–Whitney test (black dotted line) compared the two subgroups before and after LFD. Significant differences were defined as *p*-values < 0.05. The red dotted line represents the cutoff value for the Lac/Man ratio (0.030).

Figure 3 illustrates % Lac, % Man, % Suc, and the Lac/Man ratio before and after the LFD for the two patient subgroups. At the start of the diet, significant differences were found between the H-FZ and N-FZ subgroups in % Lac (*p =* 0.0058) and Lac/Man ratio (*p* = 0.033). Notably, at baseline, H-FZ patients had a mean Lac/Man ratio of 0.035, which was higher than the cutoff value of 0.030, whereas N-FZ patients did not.

After the LFD, both H-FZ and N-FZ subgroups experienced significant decreases in % Lac and the Lac/Man ratio. In the H-FZ subgroup, the Lac/Man ratio decreased by 30%, falling below the cutoff value and approaching the levels seen in N-FZ patients. At the beginning of the diet, % Suc also differed significantly between the H-FZ and N-FZ subgroups (*p* = 0.040). The LFD significantly reduced urinary Suc excretion in the H-FZ subgroup (*p* = 0.0005), whereas no significant change in % Suc was observed in the N-FZ subgroup.

Additionally, among all IBS-D patients, % Suc positively correlated with fecal zonulin levels (r = 0.32, *p* = 0.048), the n6/n3 PUFAs ratio (r = 0.44, *p =* 0.005), and IL-6 (r = 0.37, *p* = 0.002). At the conclusion of this study, no significant differences in urinary sugar levels were found between the H-FZ and N-FZ subgroups.

### 3.5. PUFAs Profile

Table 2 shows the levels of the PUFAs studied in the red blood cell membranes of IBS-D patients before and after the dietary treatment. In the whole group of IBS-D patients, *n*-6 PUFAs levels were significantly reduced after LFD (*p* = 0.010), consequently causing a reduction in the *n*-6 /*n*-3 PUFAs ratio, although not significantly. Before the diet, the H-FZ subgroup had significantly lower *n*-3 PUFAs levels (*p* = 0.008) and significantly higher *n*-6 /*n*-3 PUFAs (*p* = 0.006) and AA/EPA ratios (*p* = 0.030) than the N-FZ subgroup. Furthermore, in the H-FZ subgroup, after LFD, a significant decrease (*p* = 0.025) in *n*-6 PUFAs and a significant increase in *n*-3 PUFAs occurred (*p =* 0.043), causing a significant reduction in *n*-6 /*n*-3 PUFAs ratio (*p =* 0.002). A statistically significant decrease (*p =* 0.048) in AA/EPA ratio was also observed in this subgroup. No significant difference in PUFAs values was observed between pre- and post-treatment in the N-FZ subgroup. At the end of the treatment, no difference in the erythrocyte levels of the mentioned PUFAs was observed between the H-FZ and N-FZ subgroups. In IBS-D patients, the *n*-6/*n*-3 PUFAs ratio showed a positive correlation with fecal zonulin levels (r = 0.37, *p =* 0.023), and the AA/EPA ratio correlated positively with IL-6 serum levels (r = 037, *p =* 0.020).

### 3.6. Biomarkers of Intestinal Barrier Integrity and the Indices of Inflammation

In the overall group of IBS-D patients, serum I-FABP levels did not show significant change from the beginning to the end of this study (2.36 ± 0.37 ng/mL vs. 2.23 ± 0.39 ng/mL; pre-diet vs. post-diet). However, there were significant differences in I-FABP levels between the subgroups before the diet (*p =* 0.025). In the H-FZ subgroup, serum I-FABP decreased significantly after the diet (2.72 ± 0.76 ng/mL) compared to baseline (3.15 ± 0.71 ng/mL, *p =* 0.039). Conversely, in the N-FZ subgroup, I-FABP levels remained stable (1.65 ± 0.23 ng/mL vs. 1.79 ± 0.27 ng/mL). By the end of this study, I-FABP levels were not significantly different between the H-FZ and N-FZ subgroups. Correlations within the IBS-D group revealed that serum I-FABP levels were negatively associated with total PUFAs (r = −0.43, *p =* 0.007) and positively correlated with the AA/EPA ratio (r = 0.42, *p =* 0.009). Additionally, I-FABP levels correlated with serum IL-6 levels (r = 0.38, *p =* 0.018).

For serum DAO levels, a significant reduction was observed after the LFD (38.19 ± 0.94 ng/mL vs. 36.47 ± 0.82 ng/mL; pre-diet vs. post-diet, *p =* 0.010). This reduction was significant in both the N-FZ (37.99 ± 1.07 vs. 36.47 ± 1.27 ng/mL, *p =* 0.048) and H-FZ (39.39 ± 1.66 vs. 36.44 ± 1.06 ng/mL, *p =* 0.0061) subgroups. No significant differences in DAO levels were noted between the H-FZ and N-FZ subgroups either before or after the diet.

Table 3 presents the levels of IL-6, IL-8, IL-10, and TNF-α in IBS-D patients. IL-6 levels significantly decreased overall by the end of the diet (*p =* 0.021). Specifically, in the H-FZ subgroup, both IL-6 and IL-8 levels showed significant reductions following the treatment (*p =* 0.016 and *p =* 0.0045, respectively). No significant differences in inflammatory markers were observed between the N-FZ and H-FZ subgroups either before or after the diet.

## 4. Discussion

The results from this study on IBS-D patients indicate that those in the H-FZ subgroup displayed impaired baseline intestinal permeability, lower levels of *n*-3 PUFAs, and higher *n*-6/*n*-3 PUFA and AA/EPA ratios compared to those in the N-FZ subgroup. Following LFD, there was an improvement in s-IP and an increase in *n*-3 PUFA levels, along with reduced of IL-6 and IL-8 levels in the H-FZ subgroup. Additionally, there was a decrease in *n*-6 PUFAs, *n*-6/*n*-3 PUFA ratios, and AA/EPA ratios, indicating a shift from a pro-inflammatory to a more balanced fatty acid profile. These findings suggest that LFD may be beneficial in managing the IBS-D subgroup with impaired intestinal barriers and altered PUFA levels.

When evaluating the entire IBS-D cohort, a positive correlation between the *n*-6/*n*-3 PUFA ratio and fecal zonulin levels was observed across all participants. This study also identified elevated inflammatory markers, such as the AA/EPA ratio and IL-6, linked to intestinal barrier integrity. The presence of an altered intestinal barrier and low-grade inflammation are recognized as critical features of IBS, including IBS-D [30]. PUFAs are known for their anti-inflammatory effects in various disease states, including GI disorders [31]. While in vitro and animal studies have highlighted the relationship between PUFA levels and intestinal barrier alterations, few human studies have explored this connection [32].

Previous research [33] has shown that elevated zonulin levels, an indicator of altered s-IP, are present in both IBS-D and IBS-C patients compared to controls, with levels comparable to those seen in celiac disease, a condition known for TJ dysfunction [34]. Low-grade inflammation contributes to the impairment of s-IP, exacerbating IBS symptoms [35]. Our study found elevated AA levels and an increased AA/EPA ratio in IBS-D patients, which correlated positively with IL-6 and I-FABP, the latter being a marker of intestinal barrier integrity. These findings emphasize the low-grade inflammation characteristic of IBS-D [19]. AA, which plays a crucial role in eicosanoid synthesis, is associated with inflammation in IBS and affects gut motility and immune function [36,37]. Our results support the link between GI permeability and PUFAs, with correlations observed between PUFAs and markers of intestinal barrier function.

Dietary interventions for managing IBS have garnered increasing attention [38,39]. Our research group evaluated the role of diet in managing IBS-D patients, focusing on symptom profiles, biochemical parameters, and pathophysiological changes [19]. Consistent with the literature [40] and our previous studies [17], a 12-week LFD has shown efficacy in alleviating symptoms and maintaining intestinal barrier function and integrity. Similar benefits were observed when alternative cereals with varying gluten content, such as Tritordeum, were consumed [18]. However, the full impact of LFD on IBS-D patients remains unclear. This study specifically focused on the effects of an LFD on the PUFA composition in mature erythrocytes in IBS-D patients.

Since erythrocytes have a lifespan of approximately four months, the PUFA composition of red blood cell membranes may reflect the PUFA composition of biological tissues [19]. Our study demonstrated that an LFD significantly reduced the proportion of pro-inflammatory *n*-6 PUFAs. Additionally, LFD notably decreased serum IL-6 levels and significantly improved markers related to intestinal barrier function and integrity. Specifically, the LFD appears to influence PUFA metabolism by lowering the AA/EPA ratio, indicating a shift from a pro-inflammatory fatty acid profile to a more balanced one. While omega-3 fatty acids are known for their anti-inflammatory properties and role in maintaining intestinal barrier integrity, omega-6 fatty acids, such as AA, are associated with promoting inflammatory processes [9]. The observed changes in PUFA levels and the AA/EPA ratio align with the hypothesis that dietary interventions like LFD can modulate inflammatory pathways and enhance gut health. Overall, these results are in line with previous studies on the effects off LFD on inflammation and intestinal barrier health [17,41].

Interestingly, our study found that 47.32% of IBS-D patients had fecal zonulin levels equal to or exceeding 107 ng/mL. Patients in the H-FZ group had lower erythrocyte *n*-3 PUFA levels and a higher *n*-6/*n*-3 PUFA ratio than those in the N-FZ group. A significant difference in the AA/EPA ratio was also noted between the groups, suggesting that intestinal barrier dysfunction is linked to reduced *n*-3 PUFA levels. Lower *n*-3 PUFA levels may contribute to inflammation and negatively affect the intestinal epithelium. *N*-3 PUFAs are known for their anti-inflammatory effects and benefits for intestinal health, particularly when incorporated into epithelial cell membranes [42]. Research shows that *n*-3 PUFAs enhance intestinal barrier function by modulating TJ proteins like occludin and zonula occludens-1 [43]. They also activate the G protein-coupled receptor 120, which improves TJ stability [44]. Patients in the H-FZ subgroup exhibited higher Lac excretion and Lac/Man ratios than those in the N-FZ subgroup, indicating altered s-IP as Lac passes through the intestinal barrier via the paracellular route, reflecting TJ integrity [45]. Consistent with these findings, this study also observed elevated serum levels of I-FABP, a marker of intestinal cell damage, in the H-FZ subgroup compared to the N-FZ subgroup. Additionally, gastroduodenal permeability was altered in the H-FZ subgroup, with significantly higher urinary Suc levels than in the N-FZ subgroup.

After LFD treatment, the H-FZ subgroup showed a significant reduction in *n*-6 PUFAs, the *n*-6/*n*-3 PUFA ratio, and a substantial increase in *n*-3 PUFAs. LFD also significantly reduced IL-6 and IL-8 levels and improved GI barrier function and integrity. Notably, in the H-FZ group, the Lac/Man ratio, now considered a marker of intestinal barrier functionality, decreased by 30%, reaching a value much lower than the cutoff and similar to that of the N-FZ patients. Furthermore, significant reductions in cell integrity markers such as I-FABP and DAO were observed in the H-FZ subgroup. These results support our findings that LFD modifies PUFA composition in erythrocytes and positively affects intestinal barrier integrity and inflammation biomarkers.

The evidence suggests a strong link between intestinal barrier function and PUFAs in IBS-D. Data from the RCT LIBRE show that *n*-3 PUFAs, found in fatty fish like salmon, enhance intestinal barrier function, while saturated FAs from sweets and fast food impair it [46]. Other dietary components, such as fibers, vitamins, minerals, amino acids, and polyphenols, also play a role in maintaining intestinal barrier integrity [47]. Current and previous data [19] suggest that LFD may influence PUFA metabolism and its effects on barrier function and integrity.

This study has some limitations. The sample size was too small to draw definitive conclusions, and the study design did not include a control group, which could have provided additional insights, especially for a study focused on subjective responses. A pre-post design, while effective in demonstrating the positive impact of the intervention, cannot account for the variability of the disease over time. Moreover, this study assessed many anthropometric parameters unaffected by a placebo effect, aligning with our previous data [17]. Finally, the study design does not allow an understanding of the molecular mechanisms underlying the connection between erythrocyte membrane PUFA levels and the intestinal barrier. Future research should investigate whether specific diets high in *n*-3 PUFAs or PUFA supplementation could be considered therapeutic options for improving intestinal barrier function.

## 5. Conclusions

This study is the first to explore the link between erythrocyte PUFA content and changes in intestinal barrier function in IBS-D patients. High zonulin levels, indicative of a compromised intestinal barrier, appear to be associated with altered erythrocyte PUFA composition. The LFD restored compromised intestinal barrier function and improved the fatty acid profile in the erythrocyte membranes of this subset of IBS-D patients.

In this context, it is advisable to provide personalized dietary recommendations to maximize the diet’s benefits. These recommendations should be tailored to the patient’s symptom profile and biochemical markers, such as fecal zonulin levels, which can identify patients with compromised barrier function. A targeted dietary intervention may improve both intestinal barrier function and fatty acid metabolism.

## Figures and Tables

**Table 1:** Descriptive statistics of the anthropometric before (pre) and after (post) 12 weeks of LFD in the whole group and subgroups of IBS-D patients according to their normal (N-FZ) or high (H-FZ) fecal zonulin levels at baseline.

	Total Pre (*n* = 38)	Total Post (*n* = 38)	*p*	N-FZ Pre (*n* = 20)	N-FZ Post (*n* = 20)	*p*	H-FZ Pre (*n* = 18)	H-FZ Post (*n* = 18)	*p*
Weight (kg)	65.4 ± 1.9	62.0 ± 1.8	<0.0001	64.0 ± 2.4	61.1 ± 2.3	<0.0001	67 ± 3.0	62.9 ± 2.8	<0.0001
Height (m)	1.61 ± 0.01	1.61 ± 0.01		1.59 ± 0.01	1.59 ± 0.01		1.63 ± 0.02	1.63 ± 0.02	
BMI (kg/m^2^)	25.1 ± 0.7	23.8 ± 0.7	<0.0001	25.2 ± 0.9	24.1 ± 0.8	<0.0001	25.0 ± 1.1	23.5 ± 1.2	<0.0001
Waist circumference (cm)	79.3 ± 1.3	76.3 ± 1.7	<0.0001	79.4 ± 2.5	76.9 ± 2.1	0.0027	79.1 ± 3.1	75.6 ± 2.9	0.0001

LFD = low fermentable oligosaccharides, disaccharides, monosaccharides, and polyols (FODMAP) diet. BMI = body mass index. Data presented as the means ± SEM. Data from before and after treatment were compared using the Wilcoxon signed-rank test. The two subgroups were compared both before and after the diet using the Mann–Whitney test. Differences considered significant at *p* < 0.05.

**Table 2:** Mean percentage of red blood cell membrane PUFAs in IBS-D patients before (pre) and after (post) 12 weeks of LFD, categorized by baseline fecal zonulin levels (N-FZ or H-FZ).

Red Blood Cell Membrane PUFAs (% rel)	Total Pre (*n* = 38)	Total Post (*n* = 38)	*p*	N-FZ Pre (*n* = 20)	N-FZ Post (*n* = 20)	*p*	H-FZ Pre (*n* = 18)	H-FZ Post (*n* = 18)	*p*	n.v. (% rel.)
C20:4n6 AA)	17.42 ± 0.76	16.60 ± 0.69	ns	17.01 ± 1.32	15.92 ± 1.06	ns	17.88 ± 0.71	17.37 ± 0.84	ns	13–17
C20:5n3 (EPA)	0.80 ± 0.08	0.91 ± 0.08	ns	0.92 ± 0.12	1.04 ± 0.14	ns	0.68 ± 0.09	0.76 ± 0.07	ns	0.5–0.9
*n*-6 PUFAs	28.45 ± 0.79	26.64 ± 0.64	0.010	28.25 ± 1.25	26.75 ± 0.85	ns	28.67 ± 0.99	26.53 ± 0.99	0.025	24–34
*n*-3 PUFAs	8.35 ± 0.55	8.52 ± 0.48	ns	9.74 ± 0.81 *	8.89 ± 0.81	ns	6.81 ± 0.57 *	8.12 ± 0.51	0.043	5.7–9
Total PUFAs	36.86 ± 1.05	35.59 ± 0.85	ns	37.47 ± 1.64	35.45 ± 1.32	ns	36.19 ± 1.30	35.76 ± 1.08	ns	28–39
AA/EPA ratio	28.50 ± 3.16	23.82 ± 2.55	ns	21.26 ± 2.21 ***	20.59 ± 3.07	ns	36.54 ± 5.72 ***	27.42 ± 4.11	0.048	<15
*n*-6/*n*-3 PUFAs ratio	4.17 ± 0.55	3.54 ± 0.22	ns	3.15 ± 0.22 **	3.51 ± 0.33	ns	5.31 ± 1.10 **	3.56 ± 0.29	0.002	3–4.5

LFD = low fermentable oligosaccharides, disaccharides, monosaccharides, and polyols (FODMAP) diet; AA = arachidonic acid; EPA = eicosapentaenoic acid; PUFAs = polyunsaturated fatty acids. Data are presented as the means ± SEM. The Wilcoxon rank-sum test was used to determine the *p*-value for comparisons of pre- and post-treatment data. The Mann–Whitney test compared the two subgroups before and after the diet. Statistical significance was set at *p* < 0.05. ns = not significant. n.v.: normal values. * N-FZ pre vs. H-FZ pre, *p =* 0.008; ** N-FZ pre vs. H-FZ pre, *p =* 0.006; *** N-FZ pre vs. H-FZ pre, *p =* 0.030. At the end of this study, no significant differences in PUFAs levels were found between the H-FZ and N-FZ subgroups.

**Table 3:** The indices of inflammation (pre) and after (post) LFD in IBS-D patients as a whole group and subgroups according to their normal (N-FZ) or high (H-FZ) fecal zonulin levels at baseline.

	Total Pre (*n* = 38)	Total Post (*n* = 38)	*p*	N-FZ Pre (*n* = 20)	N-FZ Post (*n* = 20)	*p*	H-FZ Pre (*n* = 18)	H-FZ Post (*n* = 18)	*p*
IL-6 (pg/mL)	5.31 ± 0.14	5.02 ± 0.12	0.021	5.14 ± 0.20	5.02 ± 0.19	ns	5.5 ± 0.19	5.02 ± 0.16	0.016
IL-8 (pg/mL)	4.53 ± 0.39	4.12 ± 0.15	ns	4.03 ± 0.15	3.97 ± 0.12	ns	5.08 ± 0.79	4.13 ± 0.29	0.0045
IL-10 (pg/mL)	2.85 ± 0.04	2.79 ± 0.04	ns	2.77 ± 0.04	2.72 ± 0.04	ns	2.91 ± 0.06	2.91 ± 0.08	ns
TNF-α (pg/mL)	3.63 ± 0.14	3.64 ± 0.18	ns	3.44 ± 0.11	3.44 ± 0.13	ns	3.85 ± 0.27	3.86 ± 0.35	ns

LFD = low fermentable oligosaccharides, disaccharides, monosaccharides, and polyols (FODMAP) diet; IL-6 = Interleukin-6; IL-8 = Interleukin-8, IL-10 = Interleukin-10, and TNF-α = Tumor Necrosis Factor- α. Data are reported as the means ± SEM; the *p*-value was determined by the Wilcoxon rank-sum test comparing data from before and after treatment. The two subgroups were compared both before and after the diet using the Mann–Whitney test. No differences in the inflammatory profile were found between the N-FZ and H-FZ subgroups both before or after the LFD.; A *p* < 0.05 differences were considered significant; ns = not significant.

## Data Availability

The datasets used and/or analyzed during the current study are available from the corresponding author upon reasonable request.

## Abbreviations

AA	Arachidonic Acid
BMI	Body Mass Index
DAO	diamine oxidase
EPA	Eicosapentaenoic Acid
FAMEs	Fatty acid methyl esters
FAs	Fatty Acids
FID	Flame ionization detector
FODMAPs	fermentable oligosaccharides, disaccharides, monosaccharides, and polyols
GI	Gastrointestinal
H-FZ	patients with high levels of fecal zonulin
IBS	Irritable bowel syndrome
IBS-C	constipated IBS
IBS-D	diarrheal IBS,
IBS-M	mixed-pattern constipated and diarrheal IBS
IBS-SSS	IBS-Severity Scoring System
IBS-U	unclassified IBS
IDARS	IBS diet adherence report scale
I-FABP	intestinal-fatty acid-binding protein
IL-6, IL-8, IL-10	interleukins 6, 8, 10
Lac	Lactulose
LFD	Low FODMAP Diet
Man	Mannitol
N-FZ	patients with normal levels of fecal zonulin
PUFAs	Polyunsaturated fatty acids
SAT	Sugar absorption testing
s-IP	small intestinal permeability
Suc	Sucrose
TJs	Tight junctions
TNF-α	Tumor Necrosis Factor-alpha
